# Epigenetic regulation of kidney progenitor cells

**DOI:** 10.1002/sctm.19-0289

**Published:** 2020-03-12

**Authors:** Biao Huang, Zhenqing Liu, Ariel Vonk, Zipeng Zeng, Zhongwei Li

**Affiliations:** ^1^ Division of Nephrology and Hypertension, Department of Medicine and USC/UKRO Kidney Research Center, Keck School of Medicine University of Southern California Los Angeles California USA; ^2^ Department of Stem Cell Biology and Regenerative Medicine Keck School of Medicine, University of Southern California Los Angeles California USA; ^3^ Division of Oral Biology and Medicine School of Dentistry, UCLA Los Angeles California USA

**Keywords:** cell biology, developmental biology, epigenetics, kidney, stem/progenitor cell

## Abstract

The reciprocal interactions among the different embryonic kidney progenitor populations lay the basis for proper kidney organogenesis. During kidney development, three types of progenitor cells, including nephron progenitor cells, ureteric bud progenitor cells, and interstitial progenitor cells, generate the three major kidney structures—the nephrons, the collecting duct network, and the stroma, respectively. Epigenetic mechanisms are well recognized for playing important roles in organism development, in fine‐tuned control of physiological activities, and in responses to environment stimuli. Recently, evidence supporting the importance of epigenetic mechanisms underlying kidney organogenesis has emerged. In this perspective, we summarize the research progress and discuss the potential contribution of novel stem cell, organoid, and next‐generation sequencing tools in advancing this field in the future.

## Graphical abstract

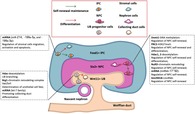

**Significance statement**

Kidney dysplasia is usually attributed to the failure of kidney progenitor cells during kidney development. Recently, great progress has been made in understanding the epigenetic mechanisms controlling the activities of kidney progenitor cells in mammalian kidney development. This perspective summarizes this progress and offers new insights into the potential application of novel technical tools in advancing this field.

## 1 INTRODUCTION

The kidney is responsible for maintaining homeostasis. It is involved in removing metabolic waste products and adjusting water, salt, and pH to maintain the homeostatic balance of fluids in mammals.^1^ In addition, the kidney also participates in the control of blood pressure through the renin‐angiotensin‐aldosterone system and secretes erythropoietin to promote erythrocyte production.^1^

During embryogenesis, the native kidney progenitor cells that give rise to the kidney include nephron progenitor cells (NPCs), ureteric bud (UB) progenitor cells (UPCs), and interstitial progenitor cells (IPCs). NPCs form nephrons, the functional units of the kidney; UPCs form the collecting duct network and the ureter that drain urine; and IPCs form various stromal cell types. Additionally, vascular progenitor cells are also present in the developing kidney to form the blood vessels.^1^, ^2^, ^3^, ^4^

Epigenetic mechanisms regulate heritable phenotype changes that do not involve alterations in the DNA sequence. In this manner, fine‐tuning of biological processes is usually achieved in response to environmental stimuli. Epigenetic mechanisms mainly include DNA methylation, histone modifications, and regulatory noncoding RNAs. To carry out these epigenetic changes, associated functional proteins serve as mediators to add or remove related epigenetic markers.^5^ Epigenetics are involved in regulating several physiological processes, such as cell differentiation,^6^ organogenesis,^7^ immune response,^8^ and organism aging.^9^

**Figure 1:**
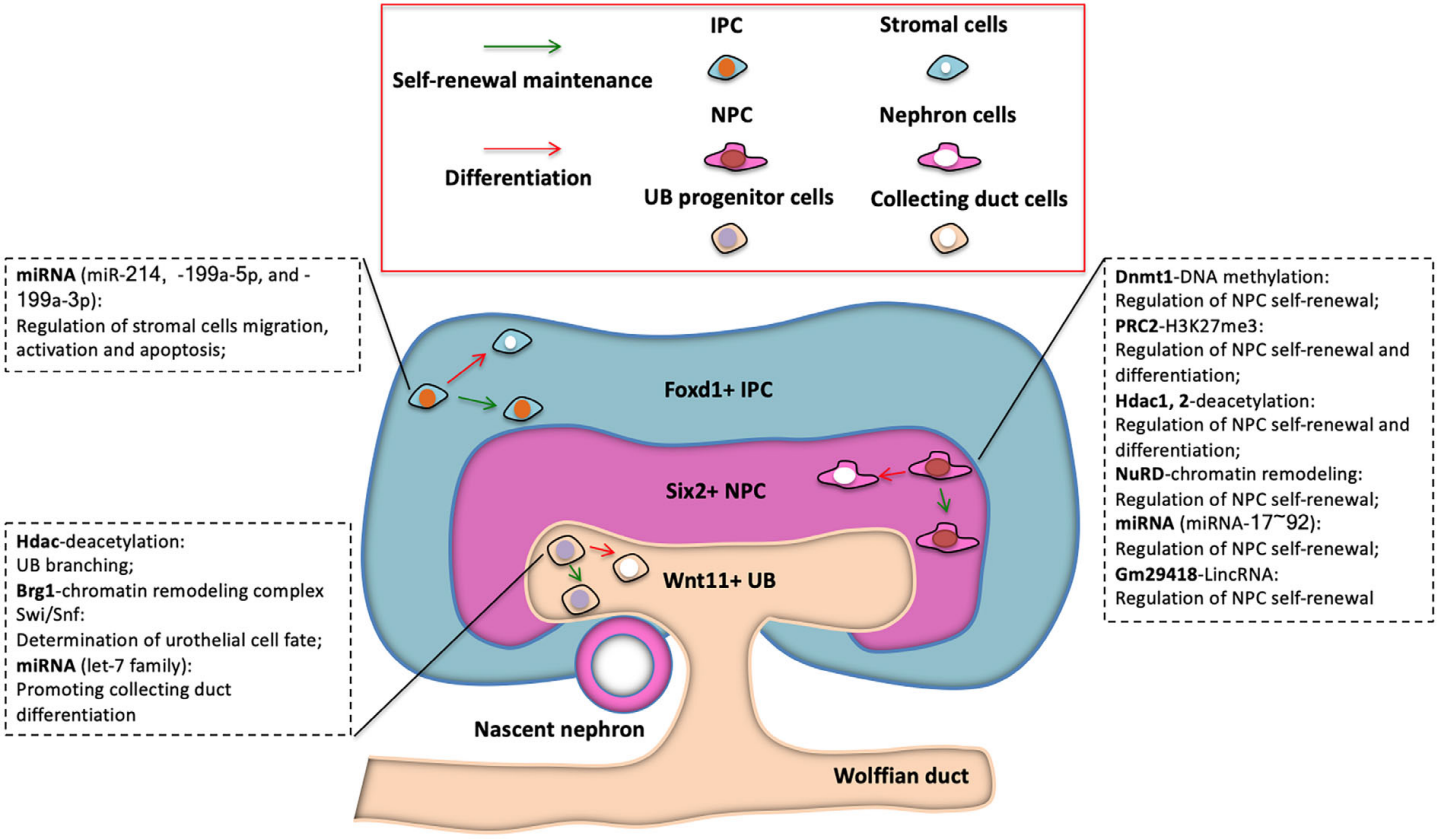
Representative scheme of epigenetic regulation of kidney progenitors within a nephrogenic niche during kidney development. Epigenetic mechanisms involved in kidney organogenesis include DNA methylation, histone acetylation, chromatin remodeling complexes, and versatile noncoding RNAs. These mechanisms are mediated by special epigenetic modifiers and play important roles in the regulation of self‐renewal maintenance and differentiation of three types of kidney progenitors during kidney development

In this perspective, we focus on discussing the recent findings of epigenetic regulation of kidney progenitor cell fates during kidney development (Figure 1).

## 2 EPIGENETIC REGULATION OF NPCs

The functional unit of the kidney is the nephron. A typical mouse kidney consists of 12 000 to 16 000 nephrons, while the human kidney has 1 000 000 nephrons on average, with significant variations among individuals.^1^ *Six2+/Cited1+* NPCs have been identified as the self‐renewing progenitor cell population that generates the main body of the nephron, including glomerulus, podocytes, proximal tubule, the loop of Henle, and distal tubule.^10^ During mouse kidney development, NPCs are specified around embryonic day 10.5 (E10.5) and are exhausted around postnatal day 2 (P2). In the humans, NPCs exist from 5 weeks to 36 weeks of the gestational age.^1^

Recently, several research groups have tried to investigate the molecular mechanisms underlying NPCs self‐renewal and differentiation during kidney development through epigenetic regulation. Using ATAC‐seq, the El‐Dahr group systematically profiled the chromatin landscape of young (E13/E16) and old (P0/P2) NPCs using fluorescence‐activated cell sorting (FACS)‐sorted primary *Six2*‐GFP + NPCs.^11^ Intrinsic differences in chromatin accessibility and enhancer landscape were uncovered. In E16 NPCs, self‐renewal genes were found to have more open chromatin at distal elements and promoters than P2 NPCs; on the contrary, poised/differentiation‐related genes showed more open chromatin in P2 NPCs as compared with the E16 NPCs. This pattern explains previous observations that old NPCs have lower barriers for differentiation^12^ and might contribute to the exhaustion of NPCs shortly after birth. The analysis of potential binding motifs in the open chromatin regions also suggested *Bach2* as a key transcription factor in transition from self‐renewal to differentiation.

NPC‐specific deletion of DNA methyltransferase 1 (*Dnmt1*) using *Six2*‐TGC (*Six2* promoter‐driven Cre); *Dnmt1* ^fl/fl^ mice led to global DNA hypomethylation and significant loss of NPCs. Consequently, *Dnmt1* deletion resulted in a remarkable reduction in nephron numbers, as well as renal hypoplasia at birth, suggesting that *Dnmt1* regulates NPC self‐renewal.^13^ Further analyses indicated that global DNA hypomethylation promotes ectopic expression of germline‐related genes and activation of endogenous retroviral elements, potentially leading to an interferon response and cell cycle inhibition, eventually interfering the NPC progenitor cell regulatory network. However, NPC‐specific knockout of *Dnmt3a* and *Dnmt3b*, the mediators of de novo DNA methylation, did not affect kidney development.

The Susztak group also generated *Dnmt1, Dnmt3a*, and *Dnmt3b* conditional knockout mice in the NPCs using *Six2* ^*Cre*^ mice. In addition, they also made NPC‐specific deletion of *Tet2*, an enzyme that mediates DNA demethylation. Consistent with the Huber group's findings, only *Dnmt1*‐conditional KO mice are indispensable for normal kidney development. However, the Susztak group reported much severe phenotype upon *Dnmt1* conditional knockout where the pups died within 24 hours after birth. Mechanistically, different from the Huber group, they did not observe significant decrease of the NPC pool in the *Dnmt1* mutant mice. They revealed that *Dnmt1* mainly work through the repression of endogenous transposable elements (TEs). The ectopic activation of TEs leads to increased IFN, RIG‐I signaling, and P53 activation in the progeny of *Six2*+ NPCs, contributing to severe kidney developmental defect.^14^

Apart from DNA methylation, another repressive epigenetic marker, trimethylation of lysine 27 on histone 3 (H3K27me3), has recently been shown to be critical in NPC self‐renewal and differentiation. Polycomb repressive complex 2 (PRC2), a chromatin‐associated methyltransferase catalyzing mono‐, di‐, and trimethylation of H3K27, was disrupted in NPCs through *Eed* KO, a key component of PRC2. Transgenic mice were generated through crossing *Eed* ^fl/fl^ KO mice with *Six2*‐TGC mice, and the phenotype changes were observed at E18.5, P0, P2, and P8. *Eed* KO resulted in smaller kidneys and premature loss of NPCs, and the investigators revealed a dual role of PRC2: it is required to maintain self‐renewal of the NPCs; during differentiation, it might also be involved in repressing self‐renewal genes, like *Six2*, to facilitate proper nephron differentiation.^15^ However, the direct target genes regulated by PRC2 remains to be determined.

Histone acetylation has been reported to regulate NPC self‐renewal and differentiation. Simultaneous deletion of both histone deacetylase 1 (*Hdac1*) and *Hdac2* in NPCs (*Hdac1* ^fl/fl^ and *Hdac2* ^fl/fl^ with *Six2*eGFPCre) led to arrest of nephrogenesis at the renal vesicle stage, renal hypodysplasia, and lethality shortly after birth. The loss of the NPC pool is responsible for the severe phenotype. Mechanistically, HDAC1 and HDAC2 are involved, as transcriptional co‐activators, in regulating NPC self‐renewal and nephrogenesis. Interactions between HDAC1/HDAC2 and core NPC transcription factors, such as SIX2, SALL1, and OSR1, can directly regulate the transcriptional programs of NPCs and renal vesicles.^16^

The regulation of NPCs by Nucleosome Remodeling and Deacetylase (NuRD) chromatin remodeling complex was revealed by crossing *Six2*‐TGC mice with *Mi2b* ^fl/fl^ mice, resulting in loss of *Mi2b* function, a key component of the NuRD complex, in the NPCs. Significant renal hypoplasia with substantial reduction in nephrons were observed. By E14.5, the expression of key NPC marker genes, *Six2* and *Cited1*, was significantly decreased and NPC proliferation capacity was reduced.^17^ The genetic interaction between *Mi2b* with *Sall1*, a key transcription factor for the specification and self‐renewal of NPCs, was revealed through the generation of heterozygous double mutants, suggesting a potential NuRD function through *Sall1*. However, the detailed mechanisms remain to be addressed to explain the strong phenotype of *Mi2b* knockout.

In view of these studies, it appears that NPC self‐renewal maintenance and proper differentiation of NPCs require a relatively repressed chromatin landscape considering that DNA hypomethylation^13^, ^14^ and histone hyperacetylation^16^ have been shown to disrupt NPC self‐renewal.

Noncoding RNAs also play an important role in epigenetic regulation. Deletion of all microRNAs (miRNAs) via conditional ablation of *Dicer* function in NPCs through crossing *Dicer* ^fl/fl^ KO mice with *Six2*‐TGC mice led to premature loss of NPCs.^18^, ^19^ Nephron progenitors largely disappeared in *Dicer* mutant kidneys by E16.5, which indicates premature depletion of NPCs. Marrone et al further revealed the role of the specific miRNA‐17~92 cluster in controlling NPC self‐renewal using conditional knockout of miR‐17~92 in the NPCs (*miR‐17~92* ^fl/fl^;*Six2*‐TGC). Deletion of microRNA‐17~92 cluster impaired NPC proliferation and reduced the number of developing nephrons.^20^ Identification of miRNA‐mRNA target interactions revealed that the potential targets of these NPC self‐renewal‐associated miRNAs are proapoptotic protein BIM (also known as BCL2L11‐BCL2‐like 11 protein)^18^ and chloride channel cystic fibrosis transmembrane conductance regulator (CFTR),^21^, ^22^ which are involved in regulation of apoptosis and proliferation in NPCs, respectively. In addition, Gm29418, a specific long noncoding RNAs (lncRNAs), was recently found to be exclusively expressed in the NPCs of the kidney, but not the other kidney cell types. *Gm29418* is located at the distal enhancer region of the *Six2* gene, suggesting its potential regulation of *Six2* at the transcription level. Knockdown and overexpression of Gm29418 in NPC‐derived cell lines showed marginal but statistically significant decrease and increase of Six2 expression, respectively.^23^ It would be interesting to investigate whether Gm29418 affects NPC self‐renewal or differentiation in vivo by conditional knockout of this lncRNA in the NPCs.

## 3 EPIGENETIC REGULATION OF UB

During mouse kidney development, UB starts to branch to form a T‐shaped structure by E11.5. The UB‐derived epithelium then undergoes 12 continuous branching steps before ceasing around 2 days after birth, finally generating the entire collecting duct system for draining of urine. *Wnt11*+ UB progenitor cells (UPCs) are positioned on the UB tips. They self‐renew to generate new UPCs at branching UB tips, while also differentiating into mature collecting duct cells, including principal cells and intercalated cells.^1^ Moreover, *p63* was identified to specifically define a subpopulation of UB tip cells, responsible for the formation of intercalated cells.^24^ The genetic determinants of UB branching have been studied extensively,^25^ while epigenetic regulations in this process are relatively less studied.

Similar to the function of HDACs in maintaining NPC self‐renewal, HDACs are also involved in UB branching morphogenesis. *Hdac1* ^fl/fl^;*Hdac2* ^fl/fl^ mice were crossed with *Hoxb7*‐CreEGFP transgenic mice to enable the conditional knockout in the ureteric epithelial of the kidney. UB‐specific deletion of both *Hdac1* and *Hdac2* resulted in bilateral renal hypodysplasia due to the impairment of canonical Wnt signaling pathway and the hyperacetylation of the tumor suppressor protein p53, which might inhibit UB cell growth and survival^26^ In addition, HDACs were also reported to participate in the renin‐angiotensin system (RAS)‐associated UB branching. HDACs are involved in regulating not only UB morphogenetic program genes, but also expression of RAS genes.^27^ Additionally, the Yu group demonstrated the significance of miRNAs in UB branching morphogenesis via UB‐specific *Dicer* deletion (*Dicer* ^fl/fl^; *Hoxb7*‐CreEGFP).^19^ Loss of *Dicer* in the UB results in a premature termination of branching morphogenesis. Let‐7 family miRNAs are expressed higher in the later‐stage UB than in the early‐stage UB, and computational analysis revealed the presence of let‐7 family miRNA binding sites for many early UB genes. These types of evidence led to the hypothesis that let‐7 family miRNAs might be involved in promoting differentiation of collecting duct via inhibiting expression of early UB genes at later developmental stages.^28^ In all these studies, *Hoxb7*‐Cre mouse strain was used. However, since *Hoxb7* is expressed in both UB tip (UPCs) and UB trunk (maturating into collecting duct), it remains unknown whether the phenotypes observed in the above studies were due to changes in the UPCs or the UPC progenies. Future studies using *Wnt11*‐Cre to mediate a specific gene manipulation in the UPCs were able to address these questions.

## 4 EPIGENETIC REGULATION OF INTERSTITIAL PROGENITOR CELLS

IPCs generate most stromal cell types in the kidney during development, including pericytes, mesangial cells, and interstitium, with *Foxd1* serving as a critical marker gene of IPCs.^29^ However, currently, there is very limited investigation into the role of epigenetics on IPCs. In 2015, Nakagawa et al reported the function of *Dicer* in renal stromal cells during kidney development.^30^ Transgenic mice were generated through crossing *Dicer1* ^fl/fl^ mice with *Foxd1*‐eGFPCre (*Foxd1*‐GC) mice. Analyses of the kidneys of the newborn pups with *Dicer* conditional knockout indicated abnormal stromal cell migration and activation, and the mice showed perinatal mortality. Moreover, the development of various other kidney structures, including nephron, collecting duct, and vasculature were disrupted, highlighting the reciprocal interactions among different progenitor populations in the developing kidney. Mechanistically, stromal cell miRNAs suppressed in stromal cells with *Dicer1* mutation are involved in migration, proliferation, and cell‐cell signaling. Additional ex vivo experiments using a human stromal cell line identified some miRNAs associated with stromal cells functions, such as *miR‐214*, *miR‐199a‐5p*, and *miR‐199a‐3p*.^31^

## 5 SUMMARY AND FUTURE DIRECTIONS

Emerging evidence indicates that various epigenetic mechanisms play important roles in controlling the cell fates of the different kidney progenitor cells. Overall, it appears that repressive epigenetic mechanisms are more involved in the maintenance of the self‐renewal status of the NPCs in vivo. Compared with the studies of NPCs, investigations into epigenetic mechanisms regulating UPCs and IPCs are still limited. Considering that the interplay among the three kidney progenitor cells is critical to kidney organogenesis, it would be important to better understand how UPC and IPC self‐renewal and differentiation are regulated by epigenetic mechanisms in the future.

**Figure 2:**
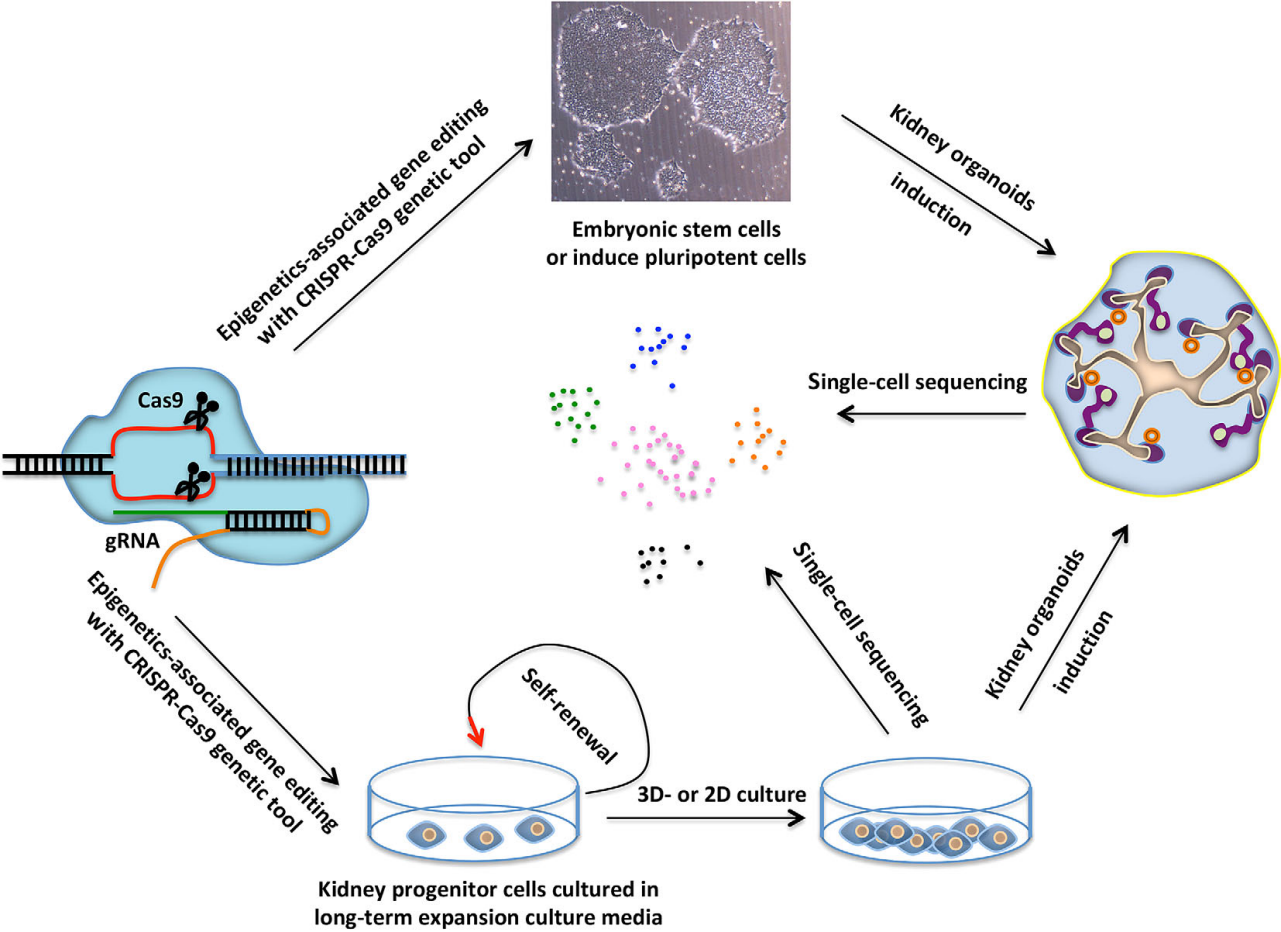
Representative scheme of new platforms to study the epigenetics of kidney progenitors based on state‐of‐the‐art technologies. in vitro kidney organoid and kidney progenitor culture systems can complement the traditional in vivo mouse genetic models in the epigenetics studies of kidney progenitors when combined with single‐cell sequencing and CRISPR‐Cas9 gene‐editing system

Most of our current knowledge in this field is obtained from studies in mouse models. Considering the differences in kidney development between mouse and human,^32^ it is imperative to investigate how the epigenetic mechanisms govern the cell fates in the human kidney progenitor cell types. Directed differentiation from human pluripotent stem cells (hPSCs) into kidney progenitor cells^33^ and kidney organoids^34^, ^35^ has emerged as a novel platform for modeling human kidney development and diseases.^36^, ^37^, ^38^, ^39^ In addition, complementing the directed differentiation from hPSCs, our group and others have developed culture conditions to expand primary mouse and human nephron progenitor cells, which could further generate nephron organoids.^40^, ^41^, ^42^ With these in vitro culture systems and gene‐editing tools, such as clustered regularly interspaced short palindromic repeats (CRISPR)–CRISPR‐associated (Cas) 9,^36^, ^43^, ^44^, ^45^ the simultaneous manipulation of multiple genes can be achieved with high efficiency, which is technically challenging and expensive to do using the traditional mouse models. In this manner, these in vitro systems can supplement the traditional mouse models in addressing the detailed epigenetic mechanisms involved in both mouse and human kidney development. The availability of large quantities of relatively pure progenitor cells will also enable novel applications that were previously impossible to do with the small number of cells isolated from the mice. For example, the genetic screening using the CRISPR‐Cas9 system^46^, ^47^ can now be done with the availability of a large number of NPCs produced in vitro. Moreover, single cell sequencing technologies have proved to be a powerful research tool recently. With a higher resolution gene expression profiling of kidney development at the single cell level, novel epigenetic mechanisms will potentially be discovered. Taken together, in the future, a better understanding of the epigenetic regulatory mechanisms will contribute to our understanding of kidney development, and the knowledge will potentially help address the pathogenesis of congenital kidney diseases that occur frequently in newborns (Figure 2).

## CONFLICT OF INTEREST

The authors declared no potential conflicts of interest.

## AUTHOR CONTRIBUTIONS

B.H.: conception and design, collection and/or assembly of data, data analysis and interpretation, manuscript writing, final approval of manuscript; Z.L.: conception and design, data analysis, and interpretation; A.V.: data analysis and interpretation; Z.Z.: collection and/or assembly of data, data analysis and interpretation; Z.L.: conception and design, manuscript writing, data analysis and interpretation, final approval of manuscript.
